# A Formative Evaluation of a Social Media Campaign to Reduce Adolescent Dating Violence

**DOI:** 10.2196/resprot.3546

**Published:** 2014-11-12

**Authors:** Danielle N Lambert, Lauren E Bishop, Stephanie Guetig, Paula M Frew

**Affiliations:** ^1^Emory University Rollins School of Public HealthDepartment of Behavioral Sciences and Health EducationAtlanta, GAUnited States; ^2^Emory University School of MedicineDepartment of MedicineDivision of Infectious DiseasesDecatur, GAUnited States

**Keywords:** adolescents, dating violence, social media, formative evaluation

## Abstract

**Background:**

The Emory Jane Fonda Center implemented the Start Strong Atlanta social marketing campaign, “Keep It Strong ATL”, in 2007 to promote the development of healthy adolescent relationships and to foster the prevention of adolescent dating abuse among 11-14 year olds.

**Objective:**

A formative evaluation was conducted to understand whether messages directed at the target audience were relevant to the program’s relationship promotion and violence prevention goals, and whether the “Web 2.0” social media channels of communication (Facebook, Twitter, YouTube, Flickr, Tumblr, and Pinterest) were reaching the intended audience.

**Methods:**

Mixed methodologies included qualitative interviews and a key informant focus group, a cross-sectional survey, and web analytics. Qualitative data were analyzed using constant comparative methodology informed by grounded theory. Descriptive statistics were generated from survey data, and web analytics provided user information and traffic patterns.

**Results:**

Results indicated that the Keep It Strong ATL social marketing campaign was a valuable community resource that had potential for broader scope and greater reach. The evaluation team learned the importance of reaching adolescents through Web 2.0 platforms, and the need for message dissemination via peers. Survey results indicated that Facebook (ranked 6.5 out of 8) was the highest rated social media outlet overall, and exhibited greatest appeal and most frequent visits, yet analytics revealed that only 3.5% of “likes” were from the target audience. These results indicate that the social media campaign is reaching predominantly women (76.5% of viewership) who are outside of the target age range of 11-14 years.

**Conclusions:**

While the social media campaign was successfully launched, the findings indicate the need for a more focused selection of communication channels, timing of media updates to maximize visibility, balancing message tone and delivery, and incorporating differentiated messaging for the target audiences. Collaboration with parents and community partners is also emphasized in order to expand the campaign’s reach and create more channels to disseminate relationship promotion and dating violence prevention messaging to the intended audience.

## Introduction

### Background

Adolescence is a developmental period in a youth’s life characterized by biological and physical changes, social role transitions, and experimentation with dating relationships [1]. According to the Youth Risk Behavior Surveillance survey, 10% of adolescent relationships result in physical violence [2]. Specific demographics are more likely to experience dating violence, including African American adolescents [2], and older adolescents (ages 16-18) who have a significantly greater risk of involvement in abusive relationships [3]. Furthermore, particularly among younger adolescents, physical aggression is perpetuated by both sexes [4-7].

###  Social Networks and Social Media

Social networking websites, termed “Web 2.0,” are principal platforms in the current state of the Internet [8]. Such websites provide a global community for individuals to contribute and respond to content [9]. In the United States, 93% of adolescents aged 12-17 access the Internet and 73-80% participate in one or more networking sites [10,11]. However, trends indicate that the youngest adolescents (ages 12-13) utilize social media at significantly lower rates. While 87% of young adolescents and 95% of older adolescents report they have a Facebook profile [12], most social media sites report less than 10% of their users are under 18.

Numerous studies suggest the effectiveness of using social media initiatives to communicate health information [13,14]. Approximately one third of adolescents ages 12-17 (31%) access the Internet for health information [15]; African American youth, girls, and lower income youth are more likely to seek health information on the Internet [14-16]. These data indicate that social media could be a highly effective tool in engaging these higher risk adolescent groups in health communication and promotion.

### Formative Evaluation of Start Strong ATL

**Figure 1:**
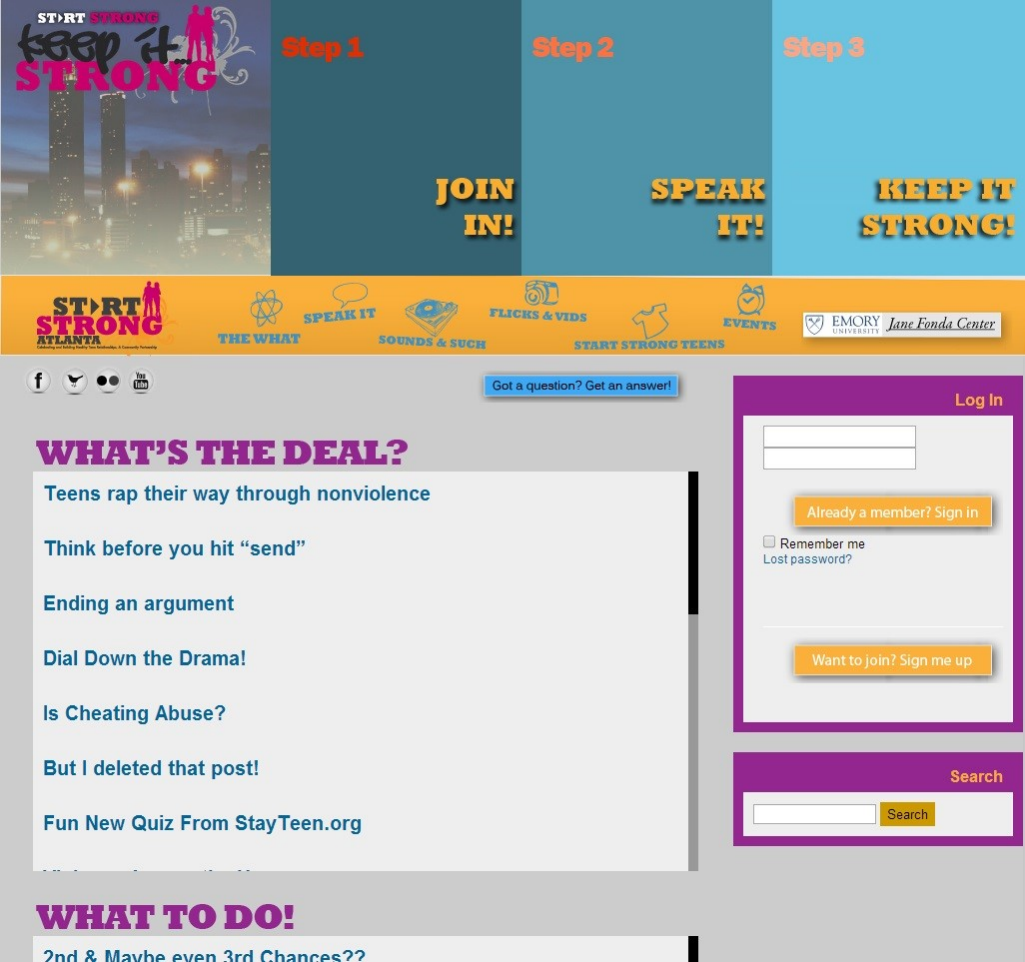
Start Strong ATL program website.

Start Strong is a national program designed to promote healthy relationships and prevent teen dating violence [17]. One of the program’s core objectives was to design and implement communication strategies to promote the program and engage youth. Start Strong Atlanta (ATL) implemented by Emory University’s Jane Fonda Center, worked with youth leaders to create and maintain a youth-focused website, striving to reduce teen dating violence among 11-14 year olds (Figure 1) [18].

**Figure 2:**
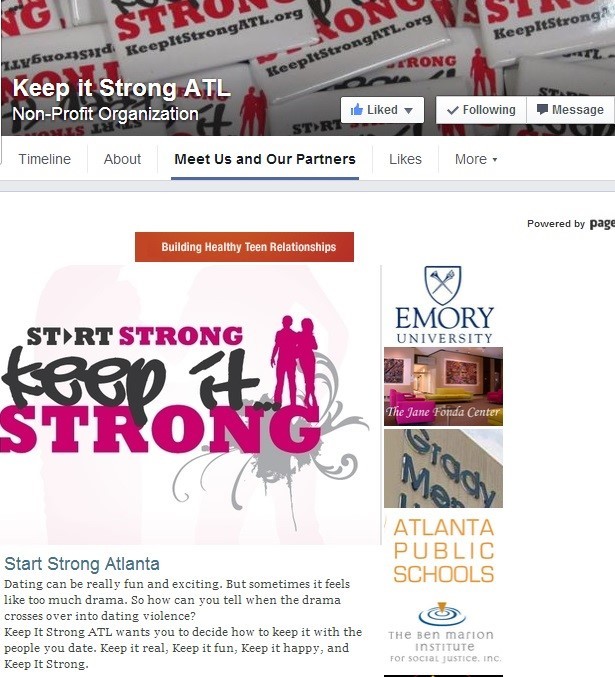
Start Strong ATL corporate Facebook page.

The purpose of the study was to examine the effectiveness of the campaign during its pilot testing period, which utilized social media platforms as dissemination to reduce teen dating violence, improve healthy adolescent relationships, and promote positive social and cultural norms [19-22]. Specifically, this evaluation sought to examine implementation challenges related to message dissemination via selected communication channels including Facebook, Twitter, YouTube, Flickr, Tumblr, and Pinterest (Figure 2) [20,23,24]. Given the lack of precedent in developing such social media campaigns, the evaluation focused on issues related to message receptiveness, source evaluation, and behavioral changes as a result of message exposure. The lack of precedent in developing social media campaigns is known for teen dating violence, but may be a more generalized phenomenon. Therefore, the results from this study will have a broader impact on the message framing and content for future online campaigns. The recommendations from this study will also aid in the future to develop successful methodologies of tailored campaigns for the young adolescent target audience in order to expand reach and awareness [25].

## Methods

### Study Recruitment

The current and previous full-time staff members (n=4) ≥18 years of age who were responsible for developing and implementing the social media campaign were recruited in person and via email from the Start Strong ATL program headquarters. These four staff members represent the medical director, project manager, former communications/media coordinator, and current communications/media coordinator. Current Start Strong ATL teen leaders (n=8) who serve as peer educators, mentors, and contributors for social media content were recruited through the Jane Fonda Center via email. Key informants, specialized in adolescent health and health communication, conducting similar social media campaigns (n=3) were recruited via email from Planned Parenthood Southeast (SE) and Emory University’s Office of Health Promotion.

A combined total of 15 respondents participated in qualitative interviews or a focus group. All 15 respondents were asked to complete an additional quantitative survey upon completing their interview. Of these respondents, 12 completed the additional quantitative survey. Google analytics were collected to examine traffic and user behavior from the Start Strong ATL program website from 2010-2012. A total of 2929 unique users from the program website were included in the analysis. Facebook Insights were also examined to isolate trends in user behavior across both the Start Strong ATL Facebook and Twitter page, which were linked to allow for simultaneous postings.

Evaluators collected data between August and December 2012, garnering comprehensive information from program staff and youth leaders, and communications personnel from community organizations. Discrete populations of respondents were used for data collection, such that all Start Strong ATL teen leaders interviewed were independent of the staff and web designers utilized to build the campaign’s website and social media sites. Additionally, all key informants interviewed were in no way contributors for or associated with the Start Strong ATL social media campaign. Participants were required to provide verbal informed consent prior to their participation. All participants were interviewed at their respective organizations to ease participant burden.

Formative research typically involves gathering data from a small number of stakeholders as the purpose of this type of evaluation is to provide insight into social media content, deployment, and use potential or its feasibility [26]. With a focus on development, this type of evaluation for social media campaigns often relies on those methods that produce results with the least amount of participant burden (eg, real-time third-party tracking data). In practice, formative research may include very small numbers of participants for primary data collection via interviews, focus groups, or surveys as a program is being created [27,28]. Capturing an adequate number of respondents needed to answer “pilot phase” social media campaigns such as Start Strong, is also weighed against the commonly held evaluation standards of feasibility, utility, propriety, and accuracy of any resulting information from the investigation [29,30].

### Data Collection

The evaluators implemented a mixed methods assessment using qualitative interviews and quantitative surveys. Semi-structured interview and discussion guides were utilized to capture qualitative data; data were audiotaped and subsequently transcribed verbatim by team members. Participant burden was restricted to 45-60 minutes for key informants, and 60-90 minutes for in-depth interviewees.

The survey was developed to garner quantifiable data about message receptiveness, source evaluation, and potential behavioral outcomes of the Keep It Strong ATL social media campaign. The survey consisted of 15 items, including 2 demographic questions indicating age and gender, and 2 open-ended qualitative questions. Participants completed the questionnaire in 10 minutes or fewer to alleviate burden.

### Analytic Strategy

The social marketing principles of product, promotion, price, and place informed the development of focus group and interview questions on the use of planned social media and its content on adolescent dating violence. Drawing upon similar formative studies, the evaluators explored questions related to the campaign (product), how it was being disseminated among target audiences (promotion), the opportunity cost of time spent online and with content (price), and the utility and feasibility of specific online venues for teen dating violence content [31-34].

Evaluators used the constant comparative method, informed by grounded theory, for qualitative analysis. Evaluators continuously compared emerging themes, concepts, and indicators [35]. Two evaluators coded each interview independently before both sets of codes were compared to assess inter-coder reliability. All coding achieved a Cohen’s kappa statistic of ≥.8 indicating a satisfactory level of agreement between the raters [36]. Emergent themes with an agreement threshold of ≥.8 among all respondents were classified as major themes. Only major themes, indicating commonality among the informants, were selected for inclusion in the results.

Quantitative data were analyzed with SPSS 20.0 to examine frequencies and assess the perceived effectiveness, reach, and adherence to the program and its objectives. Open-ended questions were compiled, coded, and analyzed according to previously stated parameters. Descriptive statistics and corresponding graphics were generated for all variables of interest.

Google analytics were also utilized to conduct oversight of website traffic since live production in November 2010 to analysis in November 2012. The software collected broad demographic information concerning users, frequency and duration of visits, and mechanisms to gain traffic. Additionally, evaluators analyzed Facebook Insights, which recorded all demographics and traffic-related information for the corporate Keep It Strong ATL Facebook page. From this source, evaluators generated descriptive tracking statistics and corresponding graphics to understand the page’s reach, specifically unique viewers of original content and “viral” spread to subsequent users.

## Results

### Summary of Results

Data indicate that the Start Strong program was effective in educating adolescents and adults about reducing dating violence and promoting healthy relationships. On average, adolescents ranked the program at 8.25 (out of 10) for reducing violence and at 8.67 for promoting healthy relationships, whereas adults’ responses averaged 7.83 for both categories. Data were compared across all collection methodologies, and major themes, with an agreement threshold among respondents of ≥0.8, were classified. The major themes addressed in this paper are Communication Channels: Technology, Timing, and Refreshing Social Media; Message Source: Tone; and Message Framing: Prevention Messaging and Content. These results were pervasive among qualitative in-depth interviewees, survey results, focus group participants, and web analytics, and were the basis for informing implications for future research.

### Characteristics of the Sample

The sample is an aggregate representation of web users, key informants from the community, teen leaders, and current and former Start Strong program staff. Broad demographic data were generated for web users (n=2929) via Google analytics and Facebook Insights. The website data indicated 43.8% of the total users were between 18 and 24 years old, and over half were female (68.8%). In-depth qualitative interviews, focus group, and quantitative survey were administered to individuals affiliated with Start Strong ATL and the broader Atlanta adolescent health promotion community, including youth leaders (n=8), key informants (n=3), and program staff members (n=4). The demographic most represented is the 15-24 age cohort (age ranges included 11-14, 15-24, 24-35, and ≥35 years), and the sample consisted of marginally more females (n=8) than males (n=7).

### Communication Channels: Technology, Timing, and Refreshing Social Media

Adolescents in the focus group expressed that they are consumers of technology and social media, particularly Twitter, Facebook, and Instagram, and recommended that the campaign continue to disseminate information to adolescents via these outlets. One youth leader stated:

Since it’s social media, and technology is like the world, since (sic) everyone interacts through social media, I think that’s a big door for teenagers to get that type of information.

Ashlee

Social media is an effective vehicle for sharing information with adolescents who use social media for daily interactions. Twitter and Facebook were the most frequently visited sites; the majority of sampled participants viewed them between daily and weekly.

**Figure 3:**
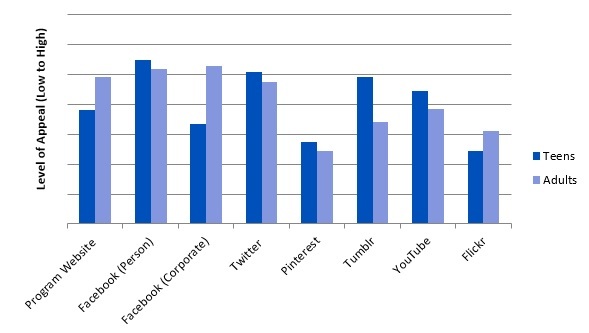
Social media site appeal for adolescents and adults as reported by program staff, (N=12).

Participants also demonstrated that Facebook and Twitter hold the greatest appeal for adolescents aged 11-14 (ranked 6.5 and 6.09 out of 8 respectively). However, Keep It Strong ATL maintains two Facebook pages: personal and corporate accounts. The personal page was ranked at 6.5 while the corporate account was ranked at 4.33. The pages with the least appeal were the Pinterest page, ranked at 3.75, and Flickr, ranked at 3.5. The program webpage was ranked at 4.82 (Figure 3).

Communication dissemination challenges, which minimized the sites’ ability to reach the target audience, were also identified. Key informants emphasized that awareness of adolescents’ social media usage is important when planning updates and posts on social media sites to achieve a high level of visibility. Carefully timing updates optimizes the target audience’s engagement. One key informant explained:

...on a Sunday night, that’s a pretty common time for people to be on Facebook, or on Twitter…between the hours of like 8:00pm and 11:00pm, you know because people are trying to procrastinate from their homework that they have due on Monday… start that hype at night when people are more likely to be on there.

Michael

Hence, key messages and programmatic content may be most effective when posted at night.

**Figure 4:**
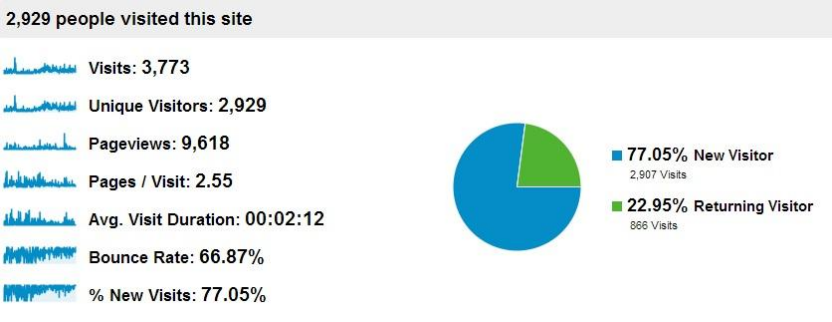
Quantity and frequency of the Start Strong website visits, 2010-2012, generated in Google Analytics.

**Figure 5:**
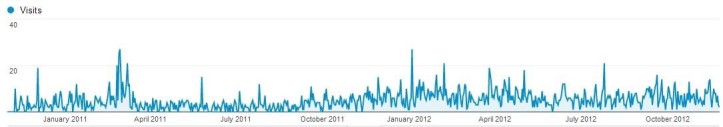
Frequency of daily visits from the Start Strong website, 2010-2012, generated in Google Analytics.

**Figure 6:**
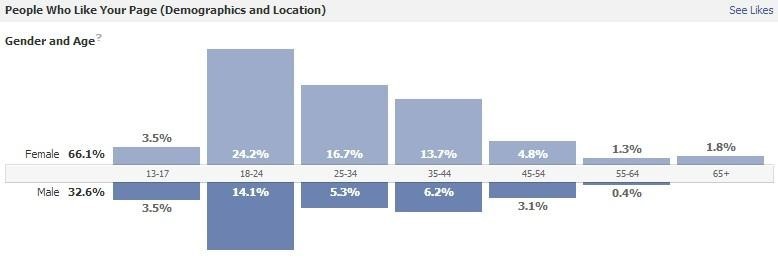
Age distribution of Facebook Likes on the Keep It Strong ATL corporate website, generated in Facebook Insights (n=223).

Analytics reflected the participants’ sentiments and provided insight into the program website traffic and engagement. From early November 2010 to late November 2012 the program’s website generated 2929 unique visitors and 9618 page views, of which 866 people visited the website multiple times (Figure 4). The average duration of each visit was 2 minutes, with 2.55 pages viewed per visit, which indicates quick page turnover among viewers. Most traffic, 77.05%, was generated by first time visitors, indicating continuous efforts to promote dissemination, but also potentially indicating that the content grew stale, given that the majority of visitors did not return to engage with updated posts. Further examination of daily visits from 2010-2012 revealed alternating periods of traffic spikes and lows (Figure 5). During initial stages of implementation, views steadily increased, followed by a sharp spike corresponding with a Public Service Announcement (PSA) video contest. Views subsequently dropped off until October 2011 when staffing changes were made. Traffic noticeably increased after this point, as the website URL was repeatedly intertwined with Facebook and Twitter postings. The most views ever reached in one day was 27, a peak which coincided with the PSA video contest; views only exceeded 20 on five additional occasions over the two years. Quantitative Facebook Insight data revealed that 66.1% of users who “like” this page were females, outside the target audience of 11-14 year olds; only 3.5% of “likes” were from the target audience (Figure 6).

### Message Source: Tone

The Keep It Strong ATL social media campaign was uniquely focused on positive messaging. Staff felt that negative content was prominent on media outlets, thus exclusively positive content was posted on program-run sites. Key informants were largely in agreement about the importance of positive messaging. However, one key informant stated:

I believe very strongly in presenting positive images and role modeling… around creating a world without violence, but saying that’s our only focus would be really diminishing of the students who need support.

Lauren

Social media users who have been victims of dating violence may need additional support. Another key informant mentioned that posted messages with the most views, “likes,” and “shares,” were humorous. She said:

Things targeting teens, we want to be serious in our messaging, but we don’t want to come off as being strict or stringent or anything, just kind of humor, a little sarcasm.

Sara

Teens respond to sarcasm and humor since they often use it in communication with each other.

Another key informant highlighted the realities of using social media to convey educational messages. The most effective tone is communicated quickly and efficiently, in short bursts. Bursts effectively gain attention without inundating with information, as this informant states:

It’s something to start awareness and hopefully gain someone’s attention in some way. And once you have them as an audience, then hopefully you can educate them after. But you’re not going to solve [dating] violence using Facebook or Twitter.

Vanessa

**Figure 7:**
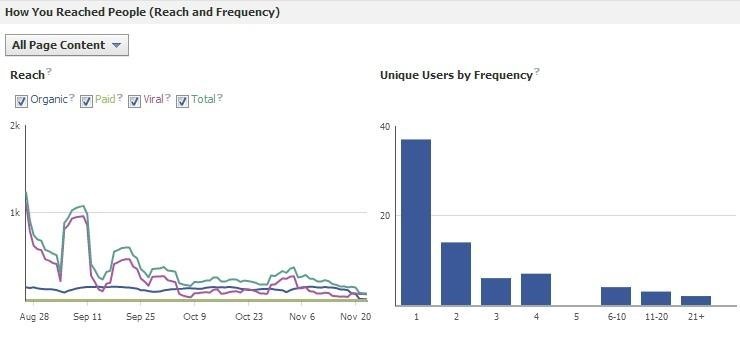
Frequency and quantity of reach per unique views for the Corporate Facebook page, generated in Facebook Insights.

Analytics highlight the results of this strategy. The site’s reach steadily declined from over 1000 users to under 200 within a 3-month span. There was one period of increased reach, corresponding to when Facebook posts were linked with Twitter, but when simultaneous posting ceased, reach began to rapidly decline (Figure 7).

### Message Framing: Prevention Messaging and Content

Key informants focused on prevention and preventive education, but also stated that prevention should not be the only campaign aim as demonstrated:

we can’t only focus on prevention, we can’t only talk about aspirations toward what healthy relationships look like, and consent… we do need to talk about students’ stories and experiences, we do need to talk about resources…that involves us working…on multiple levels for prevention.

Lauren

This suggests that primary prevention would be more effective in combination with secondary and tertiary prevention efforts, to support those who have experienced or witnessed adolescent dating violence. Stratification of user tracking data revealed limited amounts of “sharing” and reposting of Keep It Strong ATL corporate webpage and Facebook content. Females, aged 18-34 years, were the greatest proportion (59.4%) of those who reposted or shared content. This group likely found the topics of adolescent dating violence and healthy relationships relevant, motivating their engagement with the conveyed messages.

## Discussion

### Principal Findings

This formative evaluation revealed critical challenges and opportunity for the Keep It Strong ATL social media campaign [37]. During its pilot phase, the campaign was successful with engaging women and persons over 18 years of age. Yet, the data indicate that it did not reach or maintain the interest of the target audience, those in the 11-14 year old age range who remain vulnerable to adolescent dating violence [4,15]. In particular, results from qualitative and quantitative data reveal the importance of optimizing the capability of communication channels, placing emphasis on message framing and content, and selecting the right messengers or sources for success [19]. Foremost of concern for this audience is inclusion of appropriate technology, timing message dissemination, and refreshing social media content to maintain visibility and interest among adolescent viewers [15,16,19].

The results indicated that the campaign’s use of Facebook was the campaign’s most effective platform in generating viewers [38,39], particularly paired with YouTube self-generated content, links from partner pages, or relevant videos [40]; however, the majority of viewers were not representative of the target audience. Findings also indicate the importance of updating media sites during evening and weekend hours when traffic is highest and the targeted young adolescents are not in school. Yet, caution must be exercised as previous studies have linked social media use patterns to poor child sleep and health outcomes [25,41]. Adolescents in this study also emphasized the importance of timing and refreshing of messages, and improving visual messaging to promote rapid dissemination among the target audience [42]. Similar approaches have been taken with social media to reduce stigma through increased social support generated for those living with HIV in Nigeria [43]. Thus, consideration of appropriate technology use, timing of message delivery, and maintaining content currency will improve social media campaign objectives for adolescents [43,44].

Backed by Prospect Theory [45,46] and developed by psychologists Amos Tversky and Daniel Kahneman [47,48] message framing has been proposed as a potential method to promote positive health-seeking behavior [49]. Message framing in health communication typically recognizes that individuals tend to avoid risks when considering gains and prefer risks when considering losses. In this framework, gain-framed messages are developed to communicate information by emphasizing the benefits of the target health behavior. Loss-framed messages are created to emphasize the risks of not engaging in a behavior [50-54]. The campaign’s use of positively-oriented “gain-frame” messages communicate violence prevention information, emphasizing building and maintaining healthy relationships [50]. Furthermore, meta-analytic evidence from a variety of investigations indicate that gain-frame messages may be more persuasive in promoting preventive health behaviors (eg, avoiding negative relationships) [50-55]. However, results also suggest the need for message framing improvements. Positive messaging can and should remain a central focus; however, youth require context, which entails providing examples of unhealthy or inappropriate behaviors as a point of discussion and reference [5,56,57]. Furthermore, it is necessary to include resources for individuals who may have already experienced abuse or unhealthy relationships [5].

Parents and guardians often impose restrictions on preteens’ access to social media sites during this developmental stage [15]. Results indicate that the Keep It Strong ATL social media campaign did not reach its intended target audience of 11-14 year old adolescents. We believe this may be due to a lack of awareness of the existence of the program and Facebook sites, access restrictions, and messages and content that did not engage the target audience. Thus, reaching parents and other adults, such as counselors and teachers, via a theory-driven campaign approach represents an opportunity to engage the younger target audience. For example, the “Parents Speak Up National Campaign” (PSUNC) was a theory-driven multimedia parent-child campaign that achieved positive normative and outcome expectancy shifts by targeting both parents and adolescents in a “wait until older” message strategy [58]. Similarly, the Centers for Disease Control and Prevention’s (CDC) multipronged VERB media campaign targeted parents of children ages 9 to 13 years and achieved positive outcomes related to physical activity attitudes, beliefs, and behaviors which corresponded with greater than 50% of parental awareness of VERB by its third year of inception [59,60]. The formative work guiding message development, delivery, and targeting dyadic parent-child audiences evidences successful strategies that can be emulated for adolescent dating violence campaigns.

Additionally, evaluation results indicated that the program would benefit from an “Integrated Marketing Communication” (IMC) strategy that incorporates social marketing into the campaign [61]. When IMC is utilized, the campaign has greater potential to leverage the online power of schools and the broader community to increase the campaign’s relevancy and its reach to parents and adolescents. Drawing upon community capacity theory, the IMC approach has greater potential to engender support from an array of community partners meaningfully connected to the issue [62,63]. Such an opportunity presents the campaign with the greater potential to utilize all elements necessary for its success - appropriate communication channels, message sources, and salient content - to achieve program objectives.

### Limitations

We acknowledge that the sampling of a small group of participants from one southeastern city is not representative of eleven other Start Strong cities. Additionally, we recognize the potential for participatory bias, as those who agreed to participate were included and therefore may not be representative of the actual campaign population, partner organization staff, and target audiences. As this evaluation specifically examined a health campaign implemented virtually, limitations of technology must be considered. The Keep It Strong ATL social media campaign platform restricted content mapping onto the existing format, so content often grew stale and outdated, which did not encourage repeat visitors. A greater technological implication was the potential limiting impact of media sites’ policies and regulations concerning use of those in the 11-14 year old target audience. Those from the 11-14 year age range could have potentially entered older ages (invalid birthdates) to establish their accounts and bypass policies of accessibility due to age. Hence, absolute conclusions cannot be drawn about audience behavior.

### Conclusion

The Keep It Strong ATL social media campaign was successful in launching its website and Facebook sites to achieve audience engagement. Yet, results indicate the need for focused selection of communication channels, improved and resonant messaging for target audiences, and the importance of working with parents and communities to achieve broader campaign dissemination among those most vulnerable to adolescent dating violence.

## Abbreviations

CDC: Centers for Disease Control and Prevention
IMC: integrated marketing communication
PSA: public service announcement
PSUNC: Parents Speak Up National Campaign
SE: Southeast
